# HBV Infection Is an Intermediate-Risk Disease, Whereas Anaemia Is a Mild-to-Moderate Public Health Problem in Young Ghanaian Adults: A Four-Year Retrospective Analysis of Students' Medical Records

**DOI:** 10.1155/2023/9318984

**Published:** 2023-07-12

**Authors:** Samuel Amoah, Andrew Nicholas Yartey, Praise Fosu Adjei, Margaret Owusu-Akyaw, Joseph Boachie, David Larbi Simpong, Patrick Adu

**Affiliations:** ^1^University of Cape Coast Hospital Laboratory, Cape Coast, Ghana; ^2^Department of Medical Laboratory Sciences, School of Allied Health Sciences, Cape Coast, Ghana

## Abstract

**Background:**

In sub-Saharan Africa, malaria, chronic viral diseases, nutritional deficiencies, and haemoglobinopathies are common causes of anaemia. Continual surveillance data is required to situate the anaemia and infectious disease burden within a given population. This study determined the 4-year trends of anaemia, hepatitis B virus (HBV), and HCV infections and factors associated with anaemia in young Ghanaian adults.

**Methods:**

This retrospective study analysed the medical records of 21,716 fresh students at the University of Cape Coast. Data was presented as percentages and line graphs to show the yearly trends in anaemia, HBV, and HCV infections. Binary logistic regression was used to determine the increased odds of anaemia in participants.

**Results:**

Although the 4-year anaemia prevalence was 14.2% (95% CI: 0.1403–0.1498), anaemia prevalence in women and men were 24.1% (95% CI: 0.2387–0.2562) and 6.6% (95% CI:0.0616–0.0705), respectively. Anaemia prevalence consistently remained mild (males) and moderate (females) public health problem over the four-year period. Adolescents were more represented in the anaemic group (18.7% prevalence), 70.9% of them being females. The prevalence of HBV and HCV infections were 5.4% (95% CI:0.0506–0.0567) and 0.9% (95% CI: 0.0082–0.0108), respectively; only 0.1% of participants had HBV and HCV coinfection. Males were more represented in both HBV (71.2%) and HCV (63.7%) infection groups. Moreover, 15.8% of the participants who were seropositive for HBsAg self-reported having previously been vaccinated, suggesting a breakthrough infection and/or vaccine nonresponse. Furthermore, female (COR: 4.545; *p* < 0.001), teenagers (COR: 1.697; *p* < 0.001), 20–29 years (COR: 1.221; *p* = 0.035), and positive sickling slide test (COR: 1.176; *p* = 0.003) were statistically significantly associated with increased odds of anaemia.

**Conclusion:**

Intentional preventative public health campaigns regarding anaemia, HBV, and HCV infection should, respectively, target females and young adult males to increase chances of making real change in behavioural attitudes in these at-risk groups.

## 1. Background

Anaemia is a well-known haematological condition marked by decreased haemoglobin (Hb) concentration and is a global public health challenge. The prevalence of anaemia may be categorized as follows: <5%, no public health problem; 5–19.9%, mild public health problem; 20–39.9%, moderate public health problem; ≥40%, severe public health problem [1]. A recent meta-analysis reported 36.8% and 41.7% anaemia prevalence among pregnant women globally and in Africa, respectively [2]. In addition to pregnant women, anaemia may be prevalent in other high-risk groups comprising of preterm babies, infants, adolescents, lactating mothers, and reproductive-aged women. In sub-Saharan Africa, high anaemia prevalence is proposed to be associated with the concurrent prevalence of malaria, helminthic infections, and chronic viral infections, particularly HIV, tuberculosis, and genetic disorders including haemoglobinopathies [3]. Likewise, association of anaemia with hepatitis [4] and urinary tract infections [5] has been reported in some studies from other jurisdictions.

The World Health Assembly Global Nutrition has targeted 50% reduction of anaemia by the year 2025 [6]. Some key approaches adopted in the management of anaemia in sub-Saharan Africa include the prevention, accurate diagnosis and treatment of malaria [7], prevention of helminthic infections [8], reducing overall infectious disease burden, and dietary supplementation with iron, folic acid, and vitamin B12 [9]. Notwithstanding the implementation of these measures, the WHO has recommended monitoring of the population representation of anaemia prevalence via continuous surveys as a means of tracking the effectiveness of anaemia intervention control in all segments of the population [10]. Furthermore, in view of the global public health challenge posed by viral hepatitis, the WHO initiated an ambitious global health strategy on viral hepatitis that aims to attain >90% reduction in new cases of hepatitis B virus (HBV) and C (HCV) infections, as well as a 65% reduction in HBV- and HCV-related mortality by 2030 [11]. Unlike HCV, where there is no approved vaccine, HBV has an approved vaccine that has shown to be effective in preventing infection. In 2002, Ghana adopted the at-birth HBV vaccination dose as part of the expanded program of immunization to reduce the HBV infection threat. However, the vaccine coverage has been shown to be low in Africa in view of the cost implications, supply chain issues, and unreliable power supply [12]. Thus, even among those born after 2002, realistic national representative data on vaccine coverage across the country is a challenge since the at-birth HBV vaccination policy is not uniformly actualized. Even among healthcare staff in Ghana who supposedly are aware of their respective occupational risk, HBV vaccine coverage was estimated to be about 53.5% [13]. Therefore, this study sought to retrospectively analyse the medical records of freshmen university students to establish the prevalence of anaemia as well as hepatitis B and C infections as a means of acquiring empirical data to situate the burden of these conditions among the young adult population in Ghana. Since the University of Cape Coast admits students from across the breadth of the country, our current data by virtue of its large sample size provide adequate representation of the anaemia and hepatitis B and C infection situation among young adults across the country as a whole.

Recently, we reported that anaemia was a moderate public health problem that deteriorated to severe public health problem in a cohort of tertiary students [14], suggesting that Ghana is no closer to achieving the global anaemia reduction target. That study, however, did not include freshmen students who were yet to experience the stress of academic life. In order to provide a comprehensive data on the anaemia situation as well as the HBV and HCV infection burden, the present study retrospectively analysed the data from the medical screening of freshmen students at the University of Cape Coast to understand the burden of these conditions at the point of entry to the University. Additionally, the data was further explored to determine how anaemia associates with viral hepatitis B and C in these young adults. As recommended by the WHO, regular surveys and studies as reported herein represent a means of tracking the effectiveness of interventions aimed at the elimination of the socioeconomic burden of these diseases at the national level.

## 2. Methods

### 2.1. Study Design and Setting

This retrospective cross-sectional study retrieved freshmen and women students' medical screening records at the University of Cape Coast. Archival data from 2017 to 2020 were retrieved and analysed. The University of Cape Coast is an equal opportunity university meaning that admitted students come from all the regions across the country giving the data a national representation. The University of Cape Coast has a mandatory medical screening for all its freshmen and women as part of the admission requirement in order to gather data on the basic health needs of all students in the University. The routine medical screening involves hepatitis B and C testing, haemoglobin estimation, sickling slide testing, chest X-ray for evidence of tuberculosis, and urine analysis for evidence of renal pathology and/or infection. Individuals who may initially test positive to any of these conditions are referred for further evaluation and laboratory workup as per the University's policy. In view of the stigma associated with HIV infection, screening for HIV infection is not part of the testing. There were challenges with accessing the X-ray data at the radiology unit since these were manually curated. Therefore, the present report centres on haemoglobin measurements, hepatitis B and C results, urinalysis, and sickling slide test results. The period for the data collection was from July 2021 to October 2021.

### 2.2. Study Population

The study targeted students, newly admitted to study various graduate and undergraduate programs at the University of Cape Coast, Ghana. The medical screening records of a total of 21,716 were retrieved and included in the subsequent analyses. Participants belonging to the 20–39 years old category were classified as young adults in this study.

### 2.3. Inclusion and Exclusion Criteria

Fresh university students who gained admission from 2017 to 2021 and participated in the health screening process were included in the study. Fresh students who did not participate in the health screening were excluded from the current study.

### 2.4. Data Collection Procedures

Medical screening data archived at the University of Cape Coast hospital laboratory was retrieved and entered into Microsoft Excel. In addition to the demographic data, laboratory test results including haemoglobin concentration, sickling, hepatitis B test, hepatitis B vaccination status, hepatitis C test, and routine urine analysis were recorded. Haemoglobin levels were determined using a Urit-12 haemoglobin meter (URIT Medical Electronics Co. Ltd., China), whereas sickling status was determined via light microscopy after incubating participants' samples with 2% w/v sodium metabisulphite. Hepatitis B infection was established using the advanced quality one-step HBsAg test kit (InTec Products Inc., China). The detection limit of the kit as estimated by the manufacturer is 1 ng HBsAg per ml whole blood or serum/plasma sample. Evidence of hepatitis C infection was determined using the advanced quality rapid anti-HCV test kit (InTec Products Inc., China). The hepatitis B vaccination status data were recorded from the self-disclosed vaccination status by the students at the time of sample taking.

### 2.5. Data Analysis

Data entry was undertaken in Microsoft Excel 2016, and data analysis was done using SPSS version 26 (IBM SPSS, USA). All continuous variables were expressed as mean, and categorical variables were expressed as frequency and proportion. The chi-square test was employed to identify the relationships among variables. Line graphs were used to show the yearly trends in anaemia and hepatitis B and C infections. Prevalence estimations were undertaken along with a 95% confidence interval (CI) as indication of the certainty of the estimates. Additionally, binary logistic regression analysis was used to determine the factors associated with increased odds of anaemia in the participants. All statistical analyses were undertaken using the two-tail assumptions; *p* < 0.05 was taken to represent statistical significance.

## 3. Results

### 3.1. Demographic Characteristics

Overall, 56.4% of the students were males, the majority (53.2%) of whom were in the 20–29 years age group (Table 1). Furthermore, 14.2% (95% CI: 0.1403–0.1498) of the students had low haemoglobin levels per their gender-specific cut-off and were, therefore, anaemic.

### 3.2. General Health Status of Students

Table 2 shows the general health checks of the study population. Overall, 14.4% of the students (3117 of 21698) were sickling positive suggesting inheritance of haemoglobin S. The prevalence of hepatitis B virus (HBV) and hepatitis C virus (HCV) infections were 5.4% (95% CI: 0.0506–0.0567) and 0.9% (95% CI: 0.0082–0.0108), respectively. Gender-based stratification of the data showed that males were the most represented group in both HBV (71.2% vs. 28.8% females) and HCV (63.7% vs. 36.3% females) infections. Additionally, only 0.1% (22/21637) of the participants had HBV and HCV coinfection, 81.8% (18/22) of these being males. Approximately, a little over one in five of the study population (23.1%) have had hepatitis B vaccination in the past. With regards to urinary biochemical analyses, albuminuria and glycosuria were detected in 2.1% and 0.2% of freshmen, respectively.

### 3.3. Association of Hb Levels with Age, Sickling, and Gender

Hb level was shown to be significantly associated with age, sickling status, and gender with *p* values of < 0.001, 0.003, and <0.001, respectively (Table 3). Anaemia prevalence generally decreased with advancing age, with the teenage group more disproportionately (18.7%) represented in the anaemic group. Overall, females (24.1%; 95% CI: 0.2387–0.2562 vs 6.6% males; 95% CI: 0.0616–0.0705) and individuals with inherited haemoglobin S were more proportionately represented in the anaemic group. Furthermore, gender-based stratification of the anaemic teenagers revealed that 70.9% (1099/1548) were females compared to 29.1% males.

**Figure 1:**
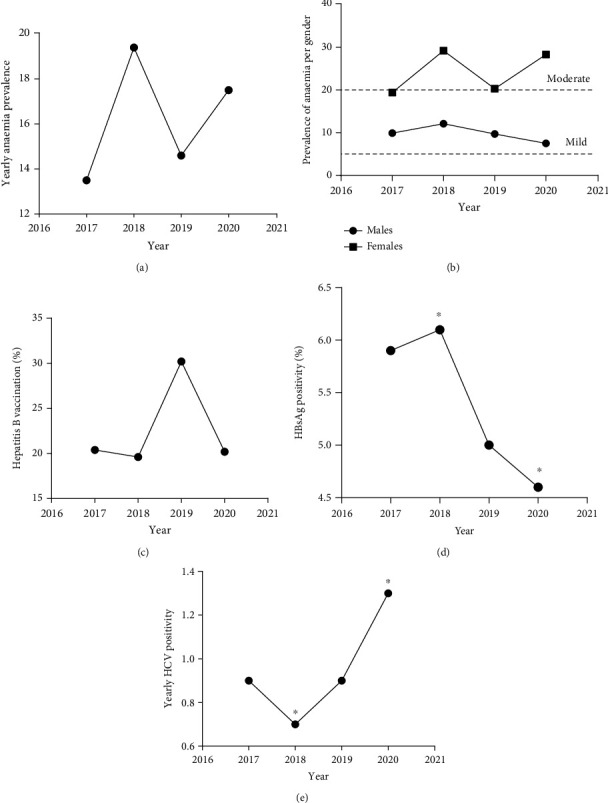
Trends in the prevalence of anaemia and hepatitis infectivity. (a) The overall yearly trend in the prevalence of anaemia over the 4-year period. (b) The 4-year anaemia prevalence per gender. (c) The proportion of individuals that were vaccinated against hepatitis B virus. (d, e) The changes in the prevalence of hepatitis B and C over the 4-year period (^∗^ indicates where significant changes in annual prevalence occurred; *p* values were estimated using chi-square to compare the annual prevalence of hepatitis B ((d) *p* = 0.0015, chi − square = 15.42) and hepatitis C ((e) *p* = 0.0126, chi − square = 10.84)).

The data was further explored for the yearly trends in the anaemia prevalence as well as hepatitis B and C infection (Figure 1). The overall prevalence of anaemia over the four-year period was a mild public health problem (5%–19.9% prevalence; Figure 1(a)). However, whereas the anaemia prevalence was consistently a mild public health problem in males over the four-year period, females registered moderate public health problem throughout the four-year period (Figure 1(b)). Moreover, hepatitis B surface antigen positivity significantly declined over the four years (Figure 1(d); *p* = 0.0015, chi − square = 15.42), whereas hepatitis C virus prevalence significantly increased after 2018 (Figure 1(e); *p* = 0.0126, chi − square = 10.84). Furthermore, over the four-year period, between 20% and 30% of the students had taken the hepatitis B vaccination (Figure 1(c)).

### 3.4. Association of HBV and HCV with Gender, Age, and Hepatitis B Vaccination

There was a significant association of HBV status with gender, age, and hepatitis B vaccination with *p* values of < 0.001, < 0.001 and, < 0.001, respectively (Table 4). Proportion-wise, more males were positive for the hepatitis B surface antigen test (6.8% males vs. 3.5% females). Also, the 40–49 years age group was disproportionately more represented in the individuals who tested positive for HBsAg test. Furthermore, of the participants who tested positive for the HBsAg test, 15.8% (181/1143) indicated having previously been vaccinated against the hepatitis B virus. When the HBV vaccination data was stratified by age, 21.3% (1771/8314), 21.7% (2484/11454), 35.0% (439/1253), 35.5% (161/454), and 41.7% (25/60), respectively, of teenagers, 20–29 years, 30–39, 40–49 years, and >49 years group self-reported having been previously vaccinated. Furthermore, over the four-year period, a significantly (*p* = 0.0326) higher proportion of males (1.1%) tested positive to HCV infection compared to females (0.8%). With respect to age, a significantly higher proportion of participants in the 20–39 years group had a higher prevalence of HCV infection (≥1.1%) compared to teenagers (0.6%) or participants in the 40–49 years group (1.1%).

The data was further explored through binary logistic regression analyses for factors that associate with anaemia in the cohort (Table 5). Participants who were female (COR: 4.545; *p* < 0.001), teenagers (COR: 1.697; *p* < 0.001), 20–29 years old (COR: 1.221; *p* = 0.035), and positive for sickling slide test (COR: 1.176; *p* = 0.003) were statistically significantly associated with increased odds of being anaemic. Additionally, although participants who were ≥50 years (COR:1.078), positive for hepatitis C infection (COR: 1.225), or have not received hepatitis B vaccination (COR: 1.026) were associated with increased odds of being anaemic, these did not reach statistical significance. However, being 40–49 years (COR: 0.427; *p* = 0.156)), or positive for hepatitis B infection (COR: 0.723; *p* = 0.003) was each associated with reduced odds of being anaemic.

## 4. Discussion

Anaemia is a serious global health problem disproportionally affecting children and women of reproductive age. The present study analysed the four-year medical records of 21,712 predominantly young adult (20–39 years old) population and estimated an overall 14.2% prevalence of anaemia. Since the university is an equal access educational institution offering admission to all people from across Ghana, our data is suggestive that anaemia is a mild public health problem among Ghanaian young adults in accordance with WHO classification. However, the overall anaemia picture is misleading since gender-based stratification of the data demonstrated that females consistently had a moderate public health anaemia problem over the four-year period compared to males who had a mild public health problem. Our logistic regression analyses provided further evidence since females had increased odds of being anaemic compared to their male counterparts. Substratification of the data revealed interesting dynamics in the anaemia picture that become masked when a global picture is presented. For example, although teenagers had significantly increased odds of being anaemic as shown by our logistic regression analysis, sex-based stratification of the data revealed that 70.9% of the teenage anaemia cases were contributed by females. This represents a serious public health concern since these are reproductive-aged women, and severe anaemia in pregnancy has been noted to have a 3.5 times greater risk of mortality from obstetric complications compared with nonanaemic pregnant women [15]. Poor dietary habit, menstrual blood loss, and lack of awareness about the importance of nutrition as well as causes and prevention of anaemia could possibly be factors underlying the data presented herein. This is alarming given the fact that the study population are newly admitted students who are at risk of developing anaemia as they begin their academic life as a consequence of the academic stress-induced poor dietary habits. For example, our previous study among a cohort of university students in their second to fourth years found that anaemia prevalence more than doubled over the course of the academic year [14]. It is interesting to note that the overall prevalence of anaemia in these freshly admitted students reported herein is similar to the baseline anaemia prevalence reported in our previous study (24.1% vs. 20.0%) among a cohort of tertiary students at the beginning of the academic year [14]. However, the anaemia prevalence worsened as it increased to 41.5% at the end of the academic year, thus becoming a severe public health problem. How well these tertiary students understand the implications of anaemia on cognitive functions and academic life, in general, is a question for future studies. What is clear, however, is that there should be a concerted university-wide public health campaign to bring home the issues of anaemia and its potential adverse impact on students' life in general.

Anaemia prevalence reported herein among these reproductive-aged women is, however, lower compared to what was reported previously (41.5% vs. 24.1%) [16]. Unlike this study, which recruited only university students, the previous study by Adu et al. recruited reproductive-aged women of diverse educational backgrounds. Although the differences in the educational background might be a factor, the present study also has a more national outlook and a larger sample size compared to the previous study that was undertaken across two of the 16 regions in Ghana. A previous cross-sectional study conducted in an Indian university estimated 55.3% of students to be anaemic, of whom 63.3% were female; thus, anaemia was found to be more common among females than males (63.3% vs. 36.7%, *p* < 0.0001) [17]. This agrees with our finding that anaemia was consistently a moderate public health problem in females compared to a mild public health problem in males. The overall prevalence of anaemia among the adolescent was found to be 18.7%, which is comparable to the anaemia prevalence estimated among teenagers in a previous study conducted in Laos where 19.4% of adolescents were found to be anaemic [18]. This teenage group also had increased odds of being anaemic in agreement with previous studies [19, 20]. The national nutrition program to control and manage anaemia by distributing a weekly iron supplementation for adolescent girls implemented by the Ministry of Health in 2018 [21] should be strengthened and extended to the male adolescent population in all 16 regions in Ghana. Even though this study showed a further reduction of the prevalence of anaemia burden in women, the prevalence of anaemia in Ghanaian women still remains a public health concern. Understanding the main causes of anaemia and interventions to address them is a critical component of any effort that aims to reduce anaemia in women and in the adolescents. However, the retrospective nature of the present study prevented us from establishing causality.

Furthermore, the current study, respectively, found a 5.4% and 09% prevalence of HBV and HCV infection as determined by seropositivity to HBsAg test and anti-HCV rapid test in these young adults. When the data was stratified age-wise, a lower prevalence (3.2%) of HBV infection was observed in the teenage group (15-19 years) of students compared to the 40–49-year-old group (20.9%) who were more represented. It is interesting to note that age-wise, the prevalence of hepatitis B infection increased with advancing age, peaking at 40–49 years, suggesting the likelihood of being infected proportionately increased with age. Noteworthily, the HCV infection prevalence followed a similar trend when the data was stratified per age, with peak incidence occurring in the 20–39 years group. The epidemiology of hepatitis B can be described in terms of the prevalence of hepatitis B surface antigen (HBsAg). In a population, it is broadly classified as high (>8% prevalence), intermediate (2%–7%), and low prevalence (<2%) [22]. In Ghana, the overall HBV prevalence is 8.36% according to a study conducted from 2015 to 2019 [23]. An earlier meta-analysis from 1995 to 2015 showed an HBV prevalence of 12.3% indicating that there was a decrease in HBV infection in Ghana [24]. Taken together with the previous studies, our study demonstrates a further reduction in the prevalence (5.4%) of HBV. Nonetheless, the results from this study confirm the categorization of Ghana as a moderate-high HBV-endemic country. Ghana started a national expanded program for hepatitis immunization in 2002 which aimed at reducing the prevalence of HBV infections, especially among adolescents [25]. Even though the 2015–2019 study had a prevalence of 14.3% HBV infection in the adolescent population, this study found a significant reduction since we estimated a 3.2% prevalence of HBV among adolescents. This may be indicative that the immunization program is gradually making an impact on the adolescents' population. However, the adult population took the greater proportion of the prevalence (7.9%). This could be due to the fact that the adults were born before the immunization program implementation. Importantly, the yearly trend analysis in our present study suggests that HBV infection prevalence declined over the four-year period which should offer hope towards achieving the WHO 2030 targets. It is interesting to note that although the HBV seropositivity increased with age, peaking at 40–49 years group, participants in 30–39 years, and 40–49 years group each had a better HBV vaccinated-to-unvaccinated ratio of 35.0% and 35.5%, respectively, compared to the <20–29-year group with 21.3% vaccination rate. HBV infection prevalence data can be used to estimate disease burden and guide health and vaccine policy [26] since the most efficient way of controlling HBV is by vaccination [27]. Interestingly, this study found that of the 1143 individuals who were hepatitis B virus infected, 15.8% (181/1143) reported having been vaccinated in the past. This is suggestive that 3.7% of the vaccinated population potentially had a breakthrough infection postvaccination, which is higher than the previously 1% or 1.3% prevalence reported among vaccinated healthcare workers in Ghana and Libya, respectively [28, 29]. Instructively, one cannot also rule out the possibility that an individual might vaccinate at a time when the person might have already been infected, albeit in the window period when evidence of the HBV is difficult to detect using the routine serology test. In view of the retrospective nature of the present study, we are not able to delineate whether this “breakthrough” infection was a case of individuals not completing the required dosage regimen or a case of nonresponders among the vaccinated individuals [30, 31]. Additionally, this data should be taken in the light of the fact that the hepatitis B infection screening was undertaken using the hepatitis surface antigen screening test kit. Since this was a retrospective data in which case participants were available to ascertain whether immunization was recent, we are unable to differentiate whether this was indeed a true breakthrough infection or that the test kit detected the HBsAg used in the manufacture of the vaccine especially in recently vaccinated individuals. What is not encouraging is the low intake of the hepatitis B vaccination since only 23.1% of participants self-reported having been previously vaccinated. This low hepatitis B vaccine coverage in Africa has, however, been reported by other studies [32–34]. Although HBV vaccination has been shown to effectively reduce the risk of infection [35], the cost of the vaccine has generally been considered as the main barrier that reduces the vaccine coverage in Africa [36]. Interestingly, our logistic regression analysis found that HBsAg positivity was significantly associated with reduced odds of anaemia in these young adults. The significance of this association, however, may require further studies to explore potential mechanisms.

It is equally important to note that the HCV prevalence were low in teenagers, but peaked among the 20–39 years age group in agreement with previous studies in Ghana [37, 38]. Also, just like HBV prevalence, HCV prevalence was higher in males relative to females. Previous studies in Ghana have reported various prevalence data among different segments of the population including a 1.5% pooled regional HCV prevalence [39], 5.2% among blood donors in the Greater Accra region [40], 0.5% among barbers in the Ashanti region [38], and 6.1% among blood donors in the Northern region [41]. Interestingly, we found an increasing trend towards increasing anti-HCV positivity over the four-year period. Although the retrospective nature of our study precluded us from establishing causality, a previous meta-analysis of 20-year HCV literature in Ghana (1995–2015) also demonstrated a similar fluctuating trend where anti-HCV seropositivity increased from 3.5% (1995–2000) to 6.9% (2001–2005) and then decreased to 2.4% (2011–2015) [39]. Although the HCV prevalence reported herein is lower than the 3% overall national 2016 prevalence reported in the previous meta-analysis [39], it will be interesting to explore the probable causes of the fluctuating HCV trends in Ghana. Our logistic regression analysis, however, showed that although anti-HCV seropositivity was associated with increased odds of anaemia, the association was not statistically significant.

This current study provides the most updated data on anaemia as well as hepatitis B and C burden in the young adult Ghanaian population since the students are from all regions of the country. Nevertheless, there were some limitations regarding an in-depth study on the causes of anaemia among the study population because it was a retrospective study, and the participants were not interrogated to find out the causes. Also, further investigation is needed to validate the positive hepatitis B infection and to investigate the number of vaccination dosage as well as the specific brands of the vaccine being used so as to make a conclusive statement on the effectiveness of the existing vaccination program. Such a future study that seeks to correlate the seroconversion rate vis-a-vis vaccine brand will be an important addition towards the assessment of vaccine effectiveness or nonresponders in Ghana considering that there is presently no established regulatory framework that controls which brands have access to the Ghanaian market. Furthermore, adequately controlled longitudinal studies may be required to understand the observed fluctuations in the anti-HCV seropositivity reported herein and other studies.

## 5. Conclusion

Although the anaemia situation was a moderate public health issue in young females in general, it was a severe public health issue in adolescent females. Young adult males had a higher predisposition to HBV and HCV infection. Intentional preventative public health campaigns regarding anaemia and HBV as well as HCV infection should, respectively, target females and young adult males to increase the chances of making real change in behavioural attitudes in these at-risk groups.

## Figures and Tables

**Table 1:** Baseline characteristics of freshmen.

	Frequency (*N*)	Percentage (%)
Gender		
Female	9461	43.6
Male	12251	56.4
Age (years)		
15–19	8314	38.6
20–29	11454	53.2
30–39	1253	5.8
40–49	454	2.1
>50	60	0.3
Hb (g/dL)		
Low	3079	14.2
Normal	18270	84.5
High	273	1.3

Hb: haemoglobin concentration; haemoglobin thresholds used were 11.5–16.5 g/dL (females) and 12.5–17.0 g/dL (males).

**Table 2:** General health status of freshmen.

Variables		Frequency (*N*)	Percentage (%)
Sickling status	Negative	18581	85.6
	Positive	3117	14.4

Hepatitis B status	Negative	20480	94.6
	Positive	1165	5.4

Hepatitis C status	Negative	21433	99.1
	Positive	204	0.9

Hepatitis B vaccination status	No	16343	76.9
	Vaccinated	4911	23.1

Hepatitis B and C coinfection	No	21615	99.9
	Yes	22	0.1

Urine albumin	Negative	21197	97.9
	Positive	455	2.1
	Trace	3	0.0

Urine glucose	Negative	21600	99.8
	Positive	52	0.2
	Trace	2	0.0

**Table 3:** Haemoglobin status, age, sickling, and gender.

	Total	HB (g/dL)			*p* value
		Low, *N* (%)	Normal, *N* (%)	High, *N* (%)	
Age (years)					
15–19	8271 (38.6)	1548 (18.7)	6609 (79.9)	113 (1.4)	<0.001
20–29	11411 (53.2)	1471 (12.8)	9804 (85.5)	136 (1.7)	
30–39	1249 (5.8)	135 (10.8)	1097 (87.8)	17 (1.4)	
40–49	452 (2.1)	53 (11.7)	393 (86.9)	6 (1.4)	
>50	60 (0.3)	3 (5.0)	57 (95.0)	0 (0.0)	
Sickling status					
Negative	18505 (85.6)	2577 (13.9)	15700 (84.8)	228 (1.2)	0.003
Positive	3105 (14.4)	501 (16.1)	2559 (82.4)	45 (1.4)	
Gender					
Female	9416 (43.7)	2273 (24.1)	6934 (73.6)	209 (2.2)	<0.001
Male	12138 (56.3)	805 (6.6)	11333 (92.9)	64 (0.5)	
Teenagers					
Female	4445 (53.7)	1099 (24.7)	3253 (73.2)	93 (2.1)	<0.001
Male	3826 (46.3)	449 (11.7)	3357 (87.7)	21 (0.6)	

*p* values were calculated using chi-square analysis comparing the respective proportions of the participants in the low, normal, and high haemoglobin categories.

**Table 4:** Hepatitis B status, age, gender, and vaccination status.

		Total	Hepatitis status		*p* value
			Negative, *N* (%)	Positive, *N* (%)	
HBV infection					
Gender	Female	9451 (43.7)	9116 (96.5)	335 (3.5)	0.001^∗∗∗^
	Male	12190 (56.3)	11360 (93.2)	830 (6.8)	
Age (years)	15–19	8299 (39.3)	8033 (96.8)	266 (3.2)	0.001^∗∗∗^
	20–29	11255 (53.2)	10681 (93.6)	574 (6.4)	
	30–39	1285 (6.1)	1130 (90.8)	155 (9.2)	
	40–49	220 (1.0)	144 (79.1)	76 (20.9)	
	≥50	93 (0.4)	55 (93.2)	38 (6.8)	
HB vaccination status	No	16288 (76.9)	15326 (94.1)	962 (5.9)	0.001^∗∗∗^
	Yes	4900 (23.1)	4719 (96.3)	181 (3.7)	

HCV infection					
Gender	Females	9406 (43.7)	9332 (99.2)	74 (0.8)	0.0326
	Males	12138 (56.3)	12008 (98.9)	130 (1.1)	
Age (years)	15–19	8254 (38.6)	8203 (99.4)	51 (0.6)	0.0009
	20–29	11365 (53.2)	11231 (98.8)	134 (1.2)	
	30–39	1240 (5.8)	1226 (98.9)	14 (1.1)	
	40–49	452 (2.1)	447 (98.9)	5 (1.1)	
	≥50	59 (0.3)	59 (100.0)	0 (0.0)	

^∗∗∗^Significant at *p* < 0.001; *p* values were calculated using chi-square analysis comparing the respective proportions of the participants in the low, normal, and high haemoglobin categories.

**Table 5:** Binary logistic regression analyses predicting factors associated with anaemia.

Variable	B	*p* value	COR	95% CI
Sex				
Female	1.515	<0.001	4.545	4.168–4.962
Male	Referent			
Age (years)				
<20	0.529	<0.001	1.697	1.408–2.045
20–29	0.200	0.035	1.221	1.14–1.470
30–39^∗^	Referent	—		
40–49	-0.851	0.156	0.427	0.132–1.383
≥50	0.075	0.662	1.078	0.770–1.510
Sickling				
Positive	0.162	0.003	1.176	1.055–1.310
Negative	Referent			
HBV infection				
Positive	-0.324	0.003	0.723	0.586–0.894
Negative	Referent			
HCV infection				
Positive	0.227	0.266	1.255	0.841–1.873
Negative	Referent			
Hep B vaccination				
No	-0.260	0.596	1.026	0.933–1.129
Yes				

^∗^In the age categories, participants within the 30–39 years age group were used as referents because anaemia prevalence was least in this group. COR: crude odds ratio.

## Data Availability

Data are not publicly available due to ethical considerations since these are the medical records of students. Data can, however, be made available upon reasonable request to the corresponding author.
